# Technological adoption and innovation in OECD and BRICS countries: A two‑way fixed effects difference‑in‑differences analysis

**DOI:** 10.1371/journal.pone.0347936

**Published:** 2026-11-04

**Authors:** Amsalu K. Addis, Lipeng Chen, Hailu Kendie Addis

**Affiliations:** 1 School of Economics and Management, Hanjiang Normal University, Shiyan, China; 2 School of Economics and Management, Nanchang University, Nanchang, China; 3 Amhara Regional Agricultural Research Institute, Bahir Dar, Ethiopia; University of Karachi, PAKISTAN

## Abstract

Understanding how technological progress unfolds across advanced and emerging economies remains central to both innovation research and development policy. This study compares the trajectories of technological adoption and innovation in OECD and BRICS countries using a two-way fixed effects difference-in-differences (DID) framework over the period 1995–2023. The analysis focuses on three core indicators: mobile cellular subscriptions, internet usage, and patent applications, and is supported by an extensive set of diagnostic checks, including partial R² contributions, residual normality and heteroskedasticity tests, and Ramsey RESET tests. Despite historically higher levels of connectivity and innovation in OECD countries, the estimated results indicate a relative slowdown in growth rates for mobile subscriptions, internet adoption, and patenting activity, with negative and statistically significant coefficients across specifications with and without control variables. On the other hand, BRICS countries show steady progress in all three outcomes, often rapid, catch‑up. These findings challenge conventional assumptions about the persistence of technological leadership in advanced economies and suggest that emerging economies are increasingly shaping global patterns of technology adoption and innovation. The results emphasize the necessity of policy frameworks that recognize heterogeneous pathways of technological progress and support both diffusion and innovation in a changing global economic landscape.

## 1. Introduction

The adoption and innovation of technology are vital for national development, contributing largely to the long-term growth of economy and societal well-being. How these processes unfold in advanced economies compared with emerging ones has profound implications for global competitiveness, sustainable development, and the distribution of prosperity. The correlation between technological adoption and innovation in transition economies is crucial to explain the varied developmental trajectories of countries of the Organization for Economic Co-operation and Development (OECD) and the coalition of the BRICS (Brazil, Russia, India, China, and South Africa) and offers a unique natural laboratory for this inquiry. While members of developed countries (OECD) typically possess deep innovation ecosystems, strong intellectual property regimes, and high‑quality digital infrastructure, BRICS countries have charted a different course: they often rely on foreign technology inflows, rapid mobile‑led connectivity expansion, and increasingly aggressive domestic innovation policies [1–4]. Understanding the interplay between these two groups can shed light on whether the global technological frontier is shifting and what that means for future growth and inequality.

A growing body of recent research has probed various facets of this comparison [5], for example, examined sustainability performance in twelve OECD economies alongside China and Brazil, finding that institutional quality moderates the effectiveness of green innovation. Scholars like [6] demonstrated that policy mixes, not merely R&D spending, drive circular economy innovation in Finland and the United Kingdom, hinting at why some OECD countries may see diminishing returns from traditional innovation support. At the regional level [7], highlighted the importance of urban agglomeration for innovation diffusion in Europe, while [8,9] documented the rising influence of environmental, social, and governance (ESG) considerations on corporate innovation in both advanced and emerging markets. Together, these contributions suggest that the innovation‑adoption nexus is shaped by a complex interplay of policy, geography, and market maturity, themes that lie at the heart of the present study.

The pace of BRICS countries’ development is rapidly increasing, yet the reliance on conventional energy (fossil fuel) is high. Shifting to renewable energy sources and eco-friendly technologies presents significant challenges [2,3]. Low innovation and outdated technological infrastructure, as well as the government policy in favor of traditional energies, hold back progress [10–12]. Contrastingly, OECD countries have impeccable advantages through better socio-economic structures, stronger governance, and higher innovation expenditures [13,14]. According to recent studies, the competitiveness models and policy environment in these regions have a significant impact on the success of technological markets [15,16].

Studies show that successful exploitation of competitive advantages in countries such as outlined in Porter’s Diamond model are likely to be effective in increasing growth by adopting new technologies [17]. Likewise, innovation thrives differently by country. OECD countries usually foster innovative concepts; nevertheless, BRICS countries commonly utilize foreign investments instead of developing them domestically [18]. Additionally, OECD countries are advantageous in their strategies of collaboration at the international level and quickly adopt innovative technologies [19]. Conversely, BRICS nations have a problem of poor investment in technological skill development training to take high-tech employment, a major disadvantage to global competitiveness [18].

Implementation of new technology has been linked with a range of socio-economic factors such as level of education, infrastructure quality, and government policies. The intertwined relations between these elements and technological developments are documented in the broad literature of economics. To illustrate, a research conducted by [20] proves that the more educated a workforce is, the more innovative potential it will have, because more educated individuals are better skilled in comprehending and implementing the innovative technologies. In addition to education, quality of infrastructure, such as the reliable internet connection, good transport services, and steady energy supply, plays a vital role in encouraging new technology and innovation [21,22]. Unfortunately, the digital divide that has existed over time makes up major challenges towards how unequal members of the population access and use technology. Researchers perceived, as the global internet penetration continues to increase, notable disparities between developed and developing economies [23]. Such disparities do not only suppress economic growth but also hamper technological transfer opportunity and partnership in innovation. Countries with high infrastructure are in better positions to enable the implementation and adoption of cutting-edge technologies than countries with old-fashioned or inadequate infrastructure.

According to a recent OECD data [24], the investments of member countries in research and development (R&D) were more than 2.5% of their GDP, which outstrips the average of BRICS countries (1.3%), according to the World Bank dataset from 2021 and 2022. Such an inequality in the investment in R&D poses serious challenges to BRICS in developing technological innovation. The lack of adequate investment in R&D is a deterrence to their ability to innovate, hence deterring the economic progress. Rapid advancement of technology and innovation has significantly affected the development of the global economy [25]. According to scholars, there is a strong interconnection between technological innovations and economic growth at different developmental stages. Although the BRICS’s GDP growth has been triggered by technological advancement, it has concurrently increased carbon emissions [2,3]. In contrast, the trend in OECD countries indicates a characteristic where technology brings the realization of economic and environmental advantages, which supports the difference in the innovation strategies of these two groups [26]. Moreover, the current BRICS innovation initiatives have more extended social consequences, affecting the employment rates and social welfare [27].

The adoption of innovative technologies is an important distinguishing factor between developed countries and the emerging ones [28–30]. The use of sophisticated technologies in enhancing productivity, increasing efficiency, and creating new markets has become a common practice by high-income nations that have resulted in long-term economic growth. On the other hand, the emerging economies might face adversities in the challenge of keeping up with them due to lack of investment in technology and innovation. Recent studies have highlighted notable differences in institutional frameworks and economic infrastructures between BRICS and OECD nations that determine their assimilation capabilities to new technologies. For example, studies on the topic carried out by [31] highlights the non-convergence in financial markets in these groups is mainly due to the differences in the size of the economy, the openness of the economy, and the level of financial freedom.

This study provides a comprehensive analysis of technological adoption and innovation disparities between OECD and BRICS countries from 1995 to 2023, employing a Two-Way Fixed Effect (TWFE) Difference-in-Differences (DID) framework. Crucially, while previous studies have tended to examine either technology adoption or innovation in isolation, we bring them together by analyzing three outcome variables: mobile cellular subscriptions, internet usage, and patent applications. Mobile subscriptions capture basic infrastructure reach, internet usage reflects actual digital participation, and patents serve as a proxy for innovation output. This trilogy allows us to ask whether convergence occurs uniformly across different rungs of the technological ladder. While many existing studies concentrate on individual factors or technology adoption rates, this research emphasizes the role of socio-economic elements, including GDP per capita, urban population, foreign investment, and unemployment, in shaping these dynamics. By integrating these variables within the context of OECD and BRICS countries, this study seeks to offer a more nuanced understanding of their collective influence on technological advancement.

In addition to the main analysis, we conduct a battery of statistical diagnostics—partial R², residual normality tests, heteroskedasticity checks, and Ramsey RESET specification tests—to ensure the robustness of our inferences. Ultimately, this study serves as a significant contribution to policy discussions and future research, emphasizing the interconnectedness of socio-economic conditions and technological progress in our changing world.

## 2. Literature review

The interaction between innovation and adoption of technology has received considerable attention recently, especially with regard to the dynamics of world economy between OECD countries and emerging economies, exemplified by the BRICS coalition. Their relationship has been a critical field of study in the understanding of economic development, technological adoption, and innovation. This section aims to summarize the main findings related to technological adoption and innovation metrics, analyze factors contribute to the processes, and provide a theoretical and empirical base to conduct a rigorous DID analysis.

### 2.1. Technological adoption and innovation metrics

#### 2.1.1. Mobile cellular subscriptions and internet usage.

Mobile cellular subscriptions and internet usage are important indicators of technological adoption. Various studies have continually indicated that the use of mobile technology is affected by income, infrastructure, regulatory framework, and competitiveness of the market [32,33]. The aggressive growth of mobile technology has impacted significantly on the way of communicating, conducting business, and education, specifically in developing economies [34]. As claimed by scholars, mobile subscriptions have soared in BRICS countries, which have expanded digital landscapes [35]. Conversely, OECD countries that feature an average of strong initial adoption rates exhibit a pivot toward the optimization of the current technologies [36]. This shift indicates the digitalization of the economy.

Another important measure of digital inclusion is the use of the internet. Research points out that the internet penetration rates are markedly higher in OECD nations because of the digital literacy programs and broadband access policies [37]. Conversely, in BRICS countries, internet usage is uneven due to lack of infrastructures in the rural areas even with government-led campaigns such as “Digital India” and “Internet Plus” campaign by India and China, respectively [38]. Literature has demonstrated that higher mobile subscription and internet penetration can boost connectivity, which results in better educational achievements, significant information access, and efficiency in different industries [39–42]. The research indicates that the availability of mobile services has been a transformative aspect among low income populations, which is consistent with the observations of [43] that shows mobile technology as a leading contributor of economic inclusion.

#### 2.1.2. Patent applications.

Patent applications are an essential measure of innovation output. They indicate the quantity and quality of innovative activities as well as the impact of these innovations. According to the theory of creative destruction developed by Schumpeter, innovation is the key to economic growth, and patents are used as the means of protection of innovator and an indicator of technological advancement [44,45]. Studies have pointed out that countries that have strong patent systems have increased degree of innovations [46]. In this regard, the comparison of patents in OECD and BRICS countries gives insights into the diverging dynamics of innovation ecosystems.

OECD countries dominate the world patent applications because of their developed research infrastructure backed by an extensive amount of R&D spending [47]. For instance, powerful intellectual property rights frameworks in countries like the US, Japan, and Germany have been used to promote innovation-intensive industries [48]. Conversely, BRICS countries have achieved significant progress in terms of patent applications in the last twenty years. With a notable support to R&D by the government, China has become one of the largest patent application countries in the world, whereas Brazil has a low-yet-innovative environment due to bureaucracy [1,49].

As noted by [5], the effectiveness of green innovation in OECD and BRICS settings depends heavily on institutional quality, while [6] show that in advanced economies like Finland and the UK, circular economy innovation responds best to integrated policy packages. At the sub‑national level [7], demonstrate that urban density and regional connectivity remain powerful engines of innovation in Europe. Meanwhile [8,9], call attention to the multidimensional nature of innovation, where firms increasingly embed environmental, social, and governance goals into their R&D strategies. These insights remind us that innovation metrics must be interpreted within their institutional and geographical contexts—an approach we adopt by explicitly controlling for urban population and economic development.

### 2.2. Factors influencing technological adoption and innovation

**Socioeconomic variables**: Several socioeconomic variables impact the success of technological adoption and innovation with urban population density turning out a crucial variable. According to research, better rates of technology adoption are frequently observed in urban locations, where resource and opportunities agglomerate contribute to increased access to mobile subscription and internet services, as well as the infrastructure required to facilitate innovation [50]. Urbanization is essential in these adoption trends, because urban centers are generally better access to numerous essential infrastructures, including educational institutions and telecommunications networks, through which technology can be more easily diffused [51].

High rates of urbanization in OECD countries have a positive relationship with technology adoption indicators (through mobile subscription) as well as innovation outcomes (through patent) [52]. The infrastructure within these urban environments facilitate a technological-friendly environment. Conversely, the pattern of urbanization in BRICS countries is diverse. For example, although China has experienced a fast urbanization process that has in turn been a major force behind technology adoption [53,54], other BRICS members, like India, have yet to conquer the challenge of large rural populace in need of relevant digital services [55]. Such difference highlights the complicated situation of technological adoption that economical growth and urbanization do not directly correlate into innovation, underscoring specific approaches to such issues are required in the less urbanized regions.

**Foreign direct investment (FDI) inflows**: FDI inflows are identified as one of the key factors in terms of innovation and transfer of technologies as well as skills [56,57], which is a crucial factor in developing an innovative ecosystem and introducing capital. Empirical evidence shows that FDI lead to host-country capabilities by promoting the transfer of sophisticated technologies through the multinational corporations into domestic markets [56,57]. Nevertheless, this improvement depends on different factors including industry, domestic regulatory systems, and domestic firm absorption capacity [58].

Research has revealed that FDI is more likely to affect technological adoptions in BRICS than in OECD countries; this disparity is mostly explained by the existing infrastructure and the development of human capital [59]. Although BRICS countries might have less developed domestic abilities initially, their high-paced development of economy may open special possibilities to succeed in a successful absorption of technology via FDI [3].

**GDP per capita:** Is one of the most important indicators of economic development and determines consumer demand of technology products and services, national investments in R&D activities, technology implementation, and innovation [60,61]. Frequent signs of higher income is the increased level of investment in technology, infrastructure, and education, which creates a favorable environment for both innovation and technological progress [62,63]. Although countries in OECD tend to have higher degree of GDP per capita, the economic situation in the BRICS group is more complicated, characterized by high level of internal disparity.

This disparity highlight a situation in which economic growth, although found in most BRICS economies, does not necessarily and directly reflect uniformly along with equivalent technology advancement in all sectors or regions in these countries [64,65]. Higher GDP per capita rates associated with OECD countries presents a more robust economic basis and make it possible to collaborate with public institutions and privates in the development of innovation projects [19]. This is usually the opposite of what is found in certain limited financial resources BRICS economies, challenging to form and maintain strong public-private relationships in the context of technological improvement and innovation programs [16].

**Unemployment rate:** Is an influential factor in defining the process of technology adoption and innovation because it determines the amount of disposable income that people can use to acquire digital equipment or services. High unemployment may also suppress innovation by restricting the number of talent people and the amount of consumer expenditure, which is essential driver of innovation [66]. Conversely, unemployment may also create entrepreneurship opportunities, as individuals seek to create their own jobs through innovative business [67]. Although OECD countries usually keep unemployment rates sufficiently low to ensure the continuity of technological advancement [68], certain BRICS countries have structural unemployment problems that reduce their ability to massively diffuse technologies [69].

## 3. Data and methodology

### 3.1. Variables and data sources

To implement difference-in-differences (DID) analysis, it is necessary to identify dependent and control variables associated with technology adoption and innovation, and collecting extensive panel information provided by authoritative sources. These sources provide detailed and standardized datasets that are required for cross-country comparisons across time. The key variables are displayed in Table 1.

**Table 1:** Variables and data sources.

Variables		Symbol	Measurement	Sources
Dependent variables (Technology and Innovation metrics)	Mobile cellular subscriptions	LnMobCell	Per 100 people	WDI 2025
	Individuals using the internet	LnInt	% of population	WDI 2025
	Patent applications, residents	LnPat	Number of patent applications	WDI 2025
Independent variables	Time variable	Post-treatment = 1	for years 2010–2023	
		Post-treatment = 0	for years 1995–2009	
	Treatment indicator	OECD = 1	If an OECD member	
		OECD = 0	If a BRICS member	
Control variables (Confounding factors)	GDP per capita	LnGDPpc	Current US$	WDI 2025
	Unemployment	LnUnEmp	Total % of total labor force (modeled ILO estimate)	WDI 2024
	Urban population	LnUrbPop	% of total population	WDI 2025
	Foreign direct investment, net inflows	LnFDI	% of GDP	WDI 2025

The paper has used a panel dataset including BRICS and 36 OECD countries, between 1995 and 2023, sourced from World Bank Development Indicators databases, to analyze the trends in technological adoption and innovation (see S1 Data, a compiled dataset in the Supporting information).

The missing dataset were solved by the use of moving average interpolation technique, ensuring accurate and robust study [70]. All variables are log-transformed (Ln) to address skewness and facilitate interpretation as elasticities.

We examine three dependent variables: mobile cellular subscriptions denote mobile technology access; internet usage represents digital connectivity; and patent applications is a proxy of output of innovation. Additionally, four control variables are include, such as urban population, usually associated with increased level of technology adoption and innovation; FDI inflows measures external investment which can impact on the innovation process; GDP per capita reflects the economic potential of adoption of new technologies; and unemployment rate illustrates the economic conditions that can influence the rates of innovation. Additionally, our choice is consistent with other documented theories of technology diffusion, e.g., Diffusion Theory by Rogers [71], and given by empirical evidence in the literature on economic models of innovation, e.g., Schumpeterian Growth Models [44,45]. Furthermore, timeline is broken down into two different periods in terms of events and phenomena that are significant globally:

(i), Pre-treatment period (1995–2009): encapsulates the significant technological advancements from the internet boom to the global financial crisis. It serves as a critical baseline for understanding the evolution of communication and business practices influenced by emerging technologies. During these years, the proliferation of the internet fundamentally altered information dissemination and economic interactions, providing a rich context for subsequent analyses. By examining this timeframe, we can identify the foundational shifts in technology adoption that set the stage for later innovations. Furthermore, this period allows for an exploration of the pre-existing conditions and trends that may impact the effectiveness of technology adoption in the subsequent post-treatment phase. This historical lens is essential for accurately assessing the causal relationships in our study, as it helps delineate the effects of external shocks, such as the financial crisis, on technological trajectories.
(ii), Post-treatment period (2010−2023): characterized by transformative technology developments and policy changes, including the widespread adoption of mobile technologies, digital innovations, the implementation of the Sustainable Development Goals (SDGs), and the unprecedented global impact of the COVID-19 pandemic. This timeframe is particularly significant for analyzing the implications of digital transformation initiatives and sustainable innovation strategies. The rise of digital innovation, mobile technology, and internet penetrations has reshaped consumer behavior and business models, while the SDGs have presented a framework for integrating sustainability into technological changes. Additionally, the COVID-19 pandemic has accelerated innovation and digital transformation across sectors, prompting a reevaluation of existing practices and policies [72]. By focusing on this period, we can critically assess how these factors have influenced technology adoption and innovation, thereby providing valuable insights into the interplay between technological advancements and socio-economic recovery efforts.

We consider OECD countries as a treatment group and BRICS countries as a control (untreated) group. This is because their basic differences are in their level of economic development, institutional structures, technological foundation and innovation systems [73,74], which make them acceptable in a DID analysis. In particular, typically, OECD nations have a higher amount of industrialization, developed systems of technological adoption, strong R&D investments, and well-developed systems of intellectual property rights [75–77]. Conversely, BRICS nations are the emerging economies at different levels of technological progress and potential to innovate and frequently falling behind the OECD countries in per capita incomes, technology diffusion infrastructure, and access to world markets [15,31,78]. These differences enable us to assess the impacts of a given membership in the OECD, which is a proxy of developed economic condition on technological uptake and innovation in the long run in comparison with the BRICS group. Incidentally, we have developed the methodological framework of the study, illustrated in Fig 1.

**Fig 1:**
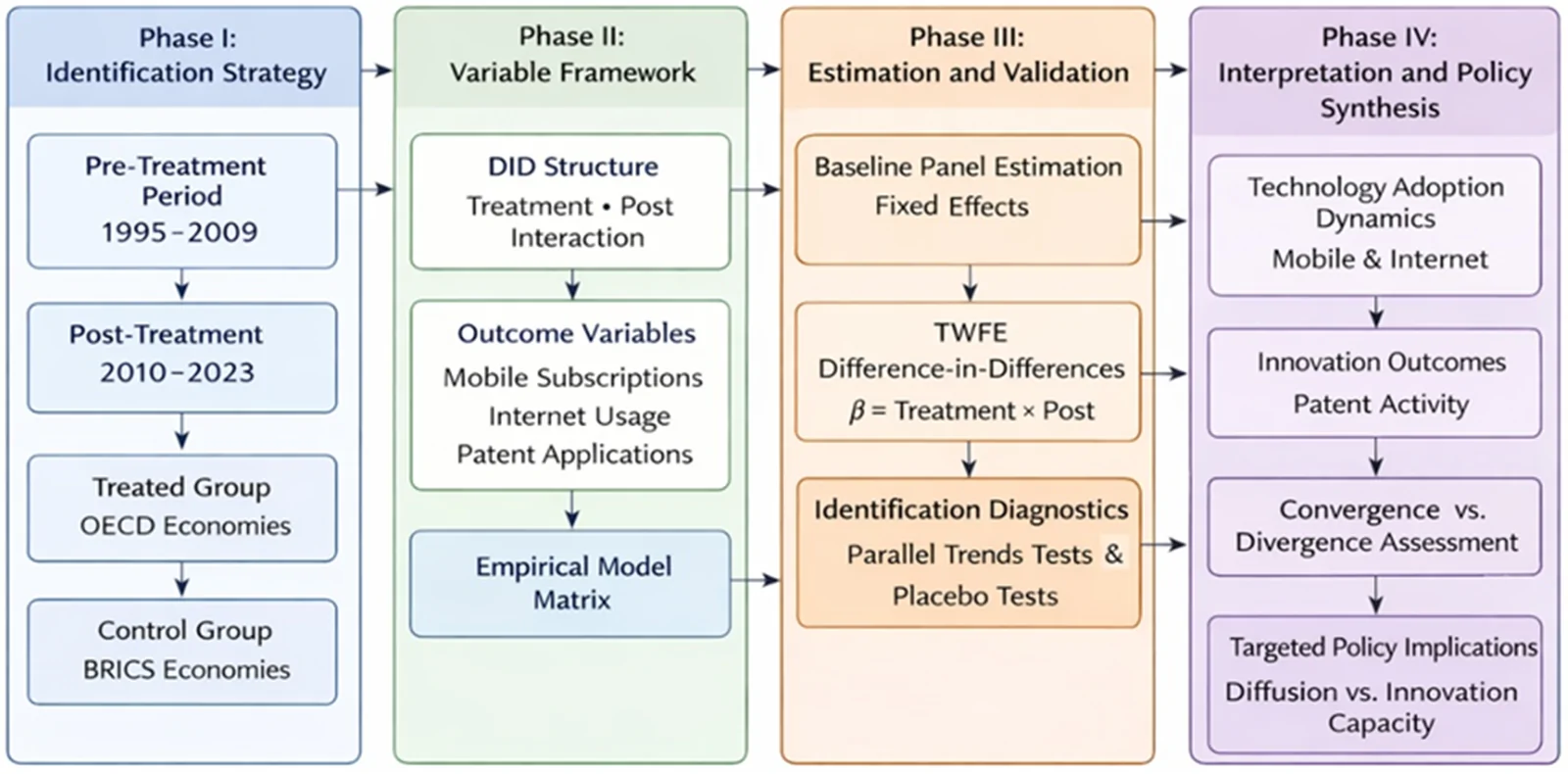
Methodological framework overview.

### 3.2. Methodology

The study used the two-way fixed effects (TWFE) models and other econometric techniques to determine how global events and changes in policies affected technology adoption and innovation in OECD and BRICS countries between 1995 and 2023. TWFE is designed to account for both multiple time periods and multiple groups by incorporating both unit and time fixed effects in the DID model [79,80]. TWFE model has seen extensive use across various research domains, shedding light on critical issues such as the effects of environmental shocks on different sectors [81,82]. It has also been instrumental in assessing the effect of political parties control of governorship on policy liberalism [83]. Similarly, it has been employed to assess the causal relationship between renewable energy generation and digitalization and renewable adoption [84]. Moreover, researchers have employed TWFE to evaluate the effect of Covid-19 pandemic [85] and effects of marriage on annual earnings [86]. The conventional TWFE framework is usually articulated through the following model specification:


$$\begin{array}{c} Y_{it}=\alpha_{i}+\lambda_{t}+\beta W_{it}+\gamma X_{it}+\varepsilon_{it} \end{array}$$ (1)

Where, $Y_{it}$ represents outcome for unit i at time t; $\alpha_{i}$ denotes unit fixed effect (country-specific), controlling for time-invariant unobserved heterogeneity specific to each unit, such as historical legal origins, geographical location, or persistent cultural attitudes toward technology; $\lambda_{t}$ represents the time fixed effect (year-specific), capturing overall macro shocks or time-specific unobserved confounders common to all units; β is the causal effect of treatment; $W_{it}$ is treatment indicator (1 if treated, 0 otherwise); $X_{it}$ represents covariates; and $\varepsilon_{it}$ is the error term.

Based on the authoritative econometric frameworks for panel data analysis to estimate the causal impact of being an OECD economy on technological indicators compared to BRICS economies, the model is specified as follows:


$$\begin{array}{c} Y_{it}=\alpha_{i}+\lambda_{t}+\beta_{\text{1}}(OECD_{i}\times Post_{t})+\sum_{j=1}^{n}\gamma_{j}X_{jit}+\varepsilon_{it} \end{array}$$ (2)

Where, $Y_{it}$ represents the dependent variables for technological adoption and innovation for country i in year t. In our study, this equation will be estimated three separate times, where Y corresponds to $LnMobCell_{it}$ mobile cellular subscriptions (per 100 people), $LnInt_{it}$ internet usage (percentage of population), and $LnPat_{it}$ patent applications (total count or per capita).

$OECD_{i}$ is a binary indicator (dummy variable) that equals 1 if the country belongs to the OECD (treated group) and 0 if it belongs to the BRICS (control group).

$Post_{t}$ is a temporal dummy variable that equals 0 for the first period (1995–2009), pre-treatment period, and 1 for the second period (2010–2023), post-treatment period.

$\beta_{\text{1}}$ represents the difference-in-differences coefficient. This is the primary parameter of interest. It measures the differential change in technological adoption/innovation for OECD countries relative to BRICS countries after the 2010 threshold.

$X_{jit}$ represents a vector of time-varying control variables included to satisfy the strict exogeneity assumption and reduce omitted variable bias. These include $LnGDPpc_{it}$ is GDP per capita (to control for the level of economic development), $LnUrbPop_{it}$ is urban population (as a proxy for infrastructure density), $LnUnEmp_{it}$ is unemployment rates (to control for labor market stability), and $LnFDI_{it}$ denotes foreign direct investment inflows (as a proxy for technology transfer).

To satisfy the requirements of the study, equation 2 is expanded into the following three specific models:

**Model 1**: Mobile cellular subscriptions (technology/infrastructure reach)


$$\begin{array}{c} LnMobCell_{it}=\alpha_{i}+\lambda_{t}+\beta_{\text{1}}(OECD_{i}\times Post_{t})+\gamma_{\text{1}}LnGDPpc_{it}+\gamma_{\text{2}}LnUrbPop_{it}+\gamma_{\text{3}}LnUnEmp_{it}+\gamma_{\text{4}}LnFDI_{it}+\varepsilon_{it} \end{array}$$ (3)

**Model 2**: Internet usage (technology/digital diffusion)


$$\begin{array}{c} LnInt_{it}=\alpha_{i}+\lambda_{t}+\beta_{\text{1}}(OECD_{i}\times Post_{t})+\gamma_{\text{1}}LnGDPpc_{it}+\gamma_{\text{2}}LnUrbPop_{it}+\gamma_{\text{3}}LnUnEmp_{it}+\gamma_{\text{4}}LnFDI_{it}+\varepsilon_{it} \end{array}$$ (4)

**Model 3**: Patent applications (innovation output)


$$\begin{array}{c} LnPat_{it}=\alpha_{i}+\lambda_{t}+\beta_{\text{1}}(OECD_{i}\times Post_{t})+\gamma_{\text{1}}LnGDPpc_{it}+\gamma_{\text{2}}LnUrbPop_{it}+\gamma_{\text{3}}LnUnEmp_{it}+\gamma_{\text{4}}LnFDI_{it}+\varepsilon_{it} \end{array}$$ (5)

This study also analyzed three other models by excluding control variables, which often termed the “Reduced Form” specification, offers significant advantages in academic study and causal inference. First, to capture the “Total Effect” of a treatment without adjusting for mechanisms that might influence it, providing insight into both direct and indirect impacts [87,88]. Second, excluding control variables helps avoid “Bad Control” bias, where including outcomes of the treatment could obscure the very effect being measured [89]. Presenting results without controls enhances transparency, demonstrating the raw correlations in the data and confirming model robustness if coefficients remain stable.

**Model 4**: Mobile cellular subscriptions


$$\begin{array}{c} LnMobCell_{it}=\alpha_{i}+\lambda_{t}+\beta_{\text{1}}(OECD_{i}\times Post_{t})+\varepsilon_{it} \end{array}$$ (6)

**Model 5**: Internet usage


$$\begin{array}{c} LnInt_{it}=\alpha_{i}+\lambda_{t}+\beta_{\text{1}}(OECD_{i}\times Post_{t})+\varepsilon_{it} \end{array}$$ (7)

**Model 6**: Patent applications


$$\begin{array}{c} LnPat_{it}=\alpha_{i}+\lambda_{t}+\beta_{\text{1}}(OECD_{i}\times Post_{t})+\varepsilon_{it} \end{array}$$ (8)

Additionally, this approach maximizes sample size by avoiding drops from missing data on control variables, strengthening statistical power. Lastly, it reduces multicollinearity among highly correlated variables, which can obscure true relationships [90], and supports a clear interpretation of the counterfactual—asking, simply, how the trajectories of OECD countries changed relative to BRICS after 2010 [91].

## 4. Results and discussions

### 4.1. Descriptive analysis

The descriptive statistics for the two blocs reinforce the motivation behind a DID design: the groups start from markedly different baselines, yet the recent trajectories suggest a narrowing gap in digital adoption and a surprising reversal in aggregate innovation output. S1 Table in the Supporting information presents descriptive statistics for variables related to technological adoption and innovation for OECD and BRICS countries.

For mobile cellular subscriptions, the OECD average (4.21) is nearly one point higher than the BRICS mean (3.32). However, the dispersion within the BRICS group is twice as large (standard deviation 2.14 vs. 1.08 for the OECD), and the minimum value of –4.83 for BRICS reflects the very early years when penetration was virtually zero in large countries like India. By contrast, the OECD series shows a tight, left‑skewed distribution consistent with a mature market where most observations cluster near saturation. Internet usage follows a similar, though less extreme, pattern: the OECD mean is about 0.95 above the BRICS mean, but the standard deviation in the BRICS group is again wider (1.28 vs. 0.98). These differences not only highlight the initial head‑start of advanced economies but also point to the rapid, uneven digital expansion that has characterized emerging markets—exactly the kind of dynamics that the study’s DID framework is designed to capture. Fig 2 presents the mean values for mobile cellular subscriptions, internet usage, and patent applications for both groups.

**Fig 2:**
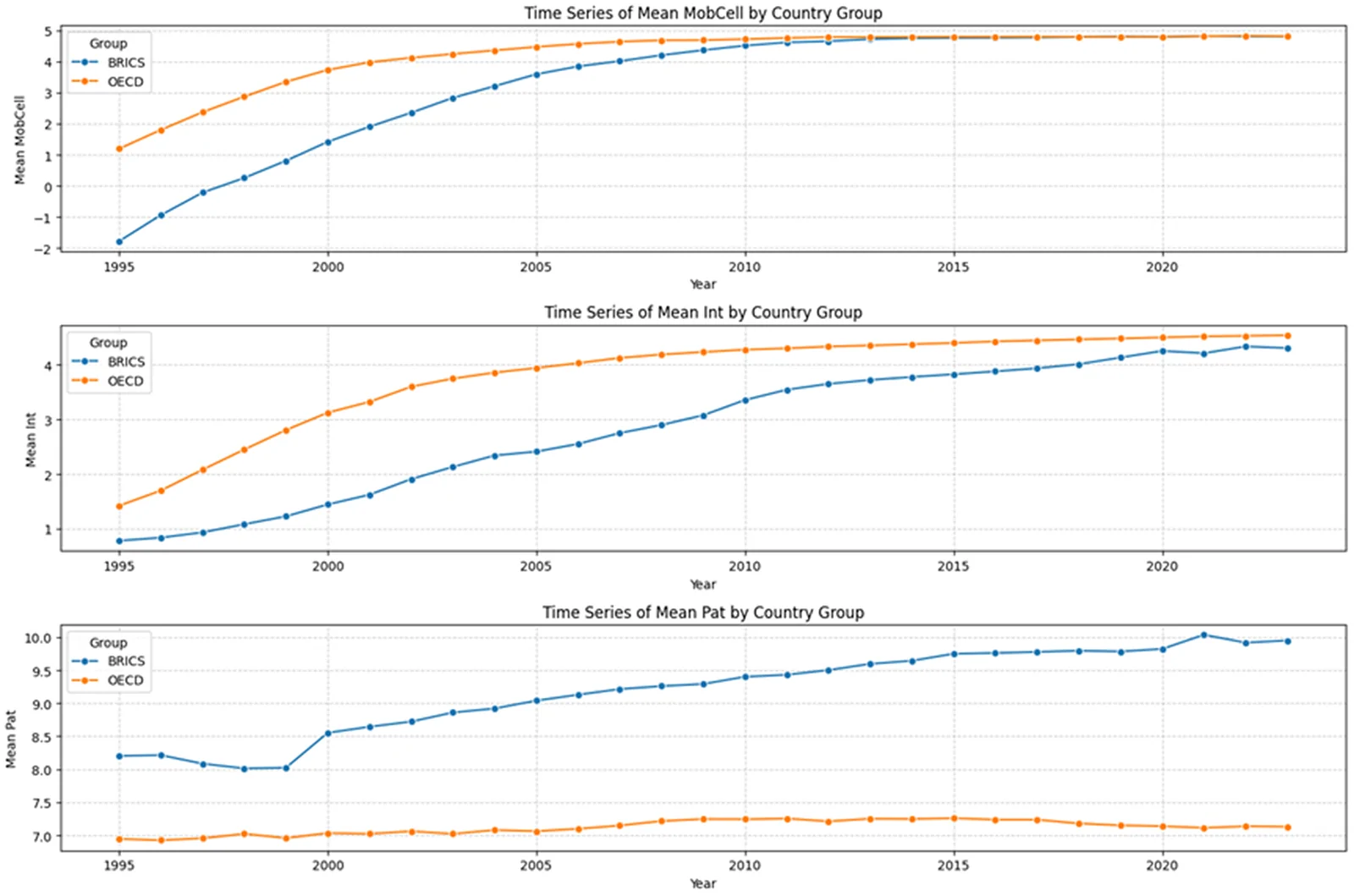
The mean of each outcome over the years (1995-2023) for both OECD and BRICS.

The most striking contrast emerges in patent applications. The BRICS group actually records a higher mean (9.18) than the OECD (7.13), despite having a substantially lower median GDP per capita. This result is primarily driven by China’s extraordinary filing activity, which pulls up the BRICS average and skews the distribution positively. The OECD distribution, while symmetric, is centered at a lower level, indicating that the bulk of advanced‑economy patenting occurs at a scale that is now modest relative to the BRICS leader. This inversion of the expected innovation hierarchy aligns with the study’s emphasis on shifting global patterns and provides preliminary support for the econometric finding of a relative slowdown in OECD patent growth after 2010.

The control‑variable trajectories reveal persistent structural gaps alongside modest convergence (S1 Fig in the Supporting information). OECD countries maintain substantially higher urbanization and GDP per capita throughout the sample, though the BRICS group’s income growth visibly steepens after 2010, creating a more favorable environment for technology (mobile and internet services) uptake [92]. FDI inflows remain virtually flat (stable) for both blocs, indicating that aggregate foreign capital is not the primary force behind the observed digital catch‑up [58]. Unemployment patterns diverge: OECD unemployment stays low with cyclical fluctuations, while BRICS unemployment is considerably more volatile, a feature that can simultaneously constrain household spending on digital services and, in certain settings, stimulate necessity‑driven entrepreneurship [67]. Together, these trends affirm the importance of controlling for economic fundamentals and help explain why basic technology adoption has been able to converge even as broad economic gaps persist.

### 4.2. DID models for each dependent variable

#### 4.2.1. Benchmark regression.

The present study investigates the differences in technological adoption and innovation between OECD (treatment) and BRICS (control) countries using a DID approach with two-way fixed effects. The analysis specifically focuses on the independent variables of mobile cellular subscriptions (LnMobCell), internet usage (LnInt), and patent applications (LnPat), while controlling for factors such as urban population (LnUrbPOP), foreign direct investment inflows (LnFDI), GDP per capita (LnGDPpc), and unemployment rates (LnUnEmp). The study covers two distinct time periods, 1995–2009 and 2010–2023, to uncover meaningful trends and derive policy implications related to technological progress and potential stagnation within these economies.

Table 2 reports the benchmark regression results on the differences in technological adoption and innovation between OECD and BRICS countries. Models (1) to (3) display the results that account for control variables, while models (4) to (6) present the results of DID regression analyses that do not incorporate control variables, with both sets of analyses controlling for country and year fixed effects. The presence of country and year fixed effects (indicated by “Country Fe” and “Year Fe”) controls for unobserved heterogeneity across countries and over time, enhancing the robustness of the estimates.

**Table 2:** Benchmark regression results.

Variables	Model 1	Model 2	Model 3	Model 4	Model 5	Model 6
	**LnMobCell**	**LnInt**	**LnPat**	**LnMobCell**	**LnInt**	**LnPat**
DID	−1.0486***	−0.6137***	−0.1732*	−1.6301***	−0.8758***	−0.9010***
	(0.1419)	(0.0661)	(0.0883)	(0.1418)	(0.0603)	(0.1247)
LnGDPpc	1.0710***	0.4827***	0.5324***			
	(0.0785)	(0.0457)	(0.0624)			
LnUnEmp	0.0952*	0.0153	0.2091***			
	(0.0512)	(0.0335)	(0.0503)			
LnUrPOP	0.6055	0.2715	4.3330***			
	(0.4911)	(0.2765)	(0.4608)			
LnFDI	0.0467***	0.0382**	−0.1079***			
	(0.0169)	(0.0168)	(0.0300)			
Constant	−9.0985***	−2.2360*	−16.1253***	4.7938***	4.0710***	7.7585***
	(2.1060)	(1.2770)	(2.1514)	(0.0571)	(0.0261)	(0.0539)
Country Fe	Yes	Yes	Yes	Yes	Yes	Yes
Year Fe	Yes	Yes	Yes	Yes	Yes	Yes
N	1,189	1,189	1,189	1,189	1,189	1,189
R²	0.8875	0.9383	0.9757	0.8579	0.9293	0.9666

**Note:** *** indicates p < 0.01, ** indicates p < 0.05, and * indicates p < 0.1; t-statistics are presented in parentheses.

**Mobile cellular subscriptions (LnMobCell)**: The DID estimate for mobile cellular subscriptions indicate that regardless of whether controlled variables are included, the regression coefficient of LnMobCell are negative and significant at p < 0.01 significance level, suggesting that mobile cellular technology has been adversely lower in OECD countries relative to BRICS nations. The differences between models (1) to (3), which account for control variables, and models (4) to (6), which do not include control variables, lie in their results: a significant negative effect, quantified at −1.0486 and −1.6301, respectively, with robust statistical significance (p < 0.01). This suggests that the growth rate of mobile cellular subscriptions in OECD countries was much less than the growth rate experienced in BRICS countries during post-treatment period, indicating stagnation in this aspect of technological adoption. Although the coefficients are still negative in both sets of analysis, −1.0486 is lower in absolute terms than −1.6301, obtained without control variables, suggesting that certain economic and demographic controls play a crucial role in elucidating the complete picture of innovation and technological adoption.

Though OECD initially had higher mobile subscriptions, its growth decreased over the years relative to BRICS. The evidence presented in [93,94] aligns with our findings. This is possibly due to slower growth rates or market saturation among OECD countries characterized by high rate of population holding mobile subscriptions could be causing decreasing marginal returns. Conversely, BRICS likely experienced rapid growth in mobile subscriptions, driven by various reasons including growing affordability, development of telecommunications infrastructure, and augmenting disposable incomes. Such a pattern is probably due to technological innovations, reduction in the costs of services, and increased acceptance of mobile devices among the consumers.

The analysis also highlight several key variables that significantly impact mobile cellular subscription. GDP per capita showed statistically significant and strong positive relationship with mobile subscriptions (coefficient 1.0710, indicating significance at the p < 0.01 threshold) and a 1% rise in GDP per capita was accompanied by a 1.071 rise in mobile subscriptions. This observation highlights the importance of interdependence between economic prosperity and availability of mobile technology; as income level increases, individuals are more possibly to adopt mobile services [92,95]. Our results are also supported by [41,96]. Additionally, the effect of unemployment rate exhibited a modest positive effect (coefficient 0.0952, p < 0.1), indicating a 1% increase in unemployment positively correlated with approximately 0.1 increase in mobile subscription rates. This correlation might also reflect the increased financial ability of the individuals to buy the mobile services when employed. Scholars articulated mobile technology adoption and access to internet can influence labor markets in various ways [97,98].

Urban population showed no significant impact on mobile subscriptions, suggesting that urban areas do not correlate with elevated mobile technology access rates [99,100]. Several factors can influence the level of this nexus, including the cost of mobile services, availability of alternative communication technologies like fixed broadband [101], and socio-economic qualities of urban residents [102,103]. FDI inflows (coefficient 0.0467, p < 0.01), implies that investment had significant direct effect in increasing subscription rates, denoting investment have necessarily be effective in increasing mobile coverage, which contradicts the findings of [104]. Scholars argue that FDI does not always have a direct relationship with mobile cellular subscriptions since it can focus on other non-telecommunication sectors [105]. The positive coefficient associated with these control variables suggests that mobile technology adoption is generally lower in OECD countries compared to BRICS countries. This finding indicates that BRICS nations may be experiencing quicker advancements in mobile technology, possibly due to different market dynamics, investment strategies, and innovative practices that can outpace the more established economies of OECD nations.

**Internet usage (LnInt)**: In our analysis of the factors influencing internet usage across OECD and BRICS countries, the benchmark regression results indicate that, regardless of whether control variables are included or excluded in the DID regression analyses, the coefficients for LnInt are negative (−0.6137 and −0.8758, respectively) and statistically significant, with p-values less than 0.01. This result emphasizes a concerning trend, while BRICS countries seem to enhance their internet reach and utilization, OECD countries are experiencing a relative decline in growth rates of internet usage during post-treatment period in both sets of analyses. This suggests that OECD countries may face challenges in maintaining their competitive edge in digital accessibility and innovation.

The results highlight several key variables that significantly impact internet adoption. GDP per capita (coefficient 0.4827, p < 0.01) showed significant positive relationship with internet usage, indicating a 1% change in GDP per capita led approximately 0.5 increase in internet usage. This discovery adds importance on the interconnection between wealth and digital technology usage; the higher the income level, the more likely to use internet services [106–108]. Similarly, FDI inflows (coefficient 0.0382, p < 0.05) suggested that foreign investment has directly or immediately facilitate increased internet access. On the other hand, unemployment rate and urban population yielded insignificant coefficient of 0.0153 and 0.2715, respectively in relation to the internet usage. Unemployment rate had positive but insignificant relationship with the internet use, indicating more people are not adopted to internet due to high unemployment rates. We find support for our findings in [97] but contrary to [108]. Similarly, urbanization progress is not necessarily accompanied by increased internet access, which contradict the findings of [109].

**Patent applications (LnPat)**: The results of the DID analysis reveal a significant negative impact on patent applications among OECD countries when compared to BRICS nations, regardless of whether control variables are included or excluded. The estimated coefficients for LnPat are −0.1732 and −0.9010, both of which are statistically significant with p-values below 0.1 and 0.01, respectively. These findings highlight a concerning decline in patent innovation within OECD countries relative to their BRICS counterparts, indicating a lag of 0.1732 and 0.9010 in patent applications during the post-treatment period [110,111].

This trend potentially points to the burgeoning influence of China in the global patent landscape, characterized by its substantial volume of patent filings [49]. Such a dynamic raises crucial questions regarding the innovative capacity of OECD economies, suggesting they may be facing stagnation in their ability to generate new technologies. In contrast, the stronger performance of BRICS countries signals a period of robust innovation, enhanced technological advancements, and increased investment in R&D. This juxtaposition underlines the need for OECD nations to reassess and reinvigorate their strategies for fostering innovation to remain competitive in an evolving global market.

The inclusion of control variables reveals critical insights into the dynamics influencing patent applications. For instance, GDP per capita demonstrates a strong positive and statistically significant relationship with patent applications, with a coefficient of 0.5324 (p < 0.01). This finding underscores the essential role economic prosperity plays in driving innovation, as a higher GDP per capita often correlates with greater resources and infrastructure dedicated to R&D [60,112,113]. In addition, the unemployment rate presents a noteworthy positive correlation with innovation, as indicated by a coefficient of 0.2091 (p < 0.01). This relationship may reflect the phenomenon where higher unemployment leads to increased innovation efforts, as individuals and organizations seek new avenues for economic activity through entrepreneurship and technological advancements. However, our findings contradict those of [97] and [98], suggesting that the impact of unemployment on innovation may be context-dependent and warrants further examination.

Moreover, urban population density correlates robustly with patent applications, with a coefficient of 4.3330 (p < 0.01). This suggests that a 1% increase in urbanization facilitates innovation by roughly 4.3, as higher population densities in urban areas may create conducive environments for collaboration, knowledge exchange, and access to resources. The evidence points to the notion that vibrant urban centers are instrumental in fostering technological advancements and driving patent activity. Conversely, FDI shows a slight negative effect on patent applications, with a coefficient of −0.1079 (p < 0.01). This finding raises important questions regarding why foreign investments do not translate into increased innovation levels in the same manner as domestic factors. Understanding this disparity could provide valuable insights into the complexities of innovation ecosystems and the varying impacts of investment sources.

#### 4.2.2. Extended diagnostic tests and robust inference.

The two‑way fixed effects (TWFE) model serves as the backbone of the analysis, but its credibility rests on the validity of several underlying assumptions [114]. Table 3 (Panel A) reports a series of diagnostic tests applied to the residuals of the level specification. The Shapiro‑Wilk test rejects the null of normally distributed errors for all three outcome variables (p < 0.001), while the Breusch‑Pagan test points to significant heteroskedasticity (p < 0.001). The Breusch‑Godfrey test for first‑order serial correlation also produces p‑values indistinguishable from zero, indicating that the errors are not independently distributed over time. Finally, the Ramsey RESET test raises concerns about functional form misspecification in the mobile subscription and patent equations (p < 0.001), although the internet usage specification fares somewhat better (p = 0.0004). Such patterns are not uncommon in macro‑panel studies where the dependent variables are highly persistent and cross‑country heterogeneity is pronounced [88,115].

**Table 3:** Integrated table with Panel A (Level model diagnostics) and Panel B (Growth model diagnostics).

Panel A: Level model diagnostics				
	**Normality p**	**Breusch-Pagan p**	**Breusch-Godfrey p**	**RESET p**
LnMobCell	0.0000	0.0000	0.0000	0.0000
LnInt	0.0000	0.0000	0.0000	0.0004
LnPat	0.0000	0.0000	0.0000	0.0000
**Panel B: Growth (first-differenced) model diagnostics**				
	**Normality p**	**Breusch-Pagan p**	**Breusch-Godfrey p**	**RESET p**
LnMobCell	0.0000	0.0000	0.0000	0.4586
LnInt	0.0000	0.0000	0.7358	0.4015
LnPat	0.0000	0.0866	0.4235	0.0453

To assess whether these violations stem from a genuine misspecification or simply from the time‑series properties of the variables, we re‑estimated all models in first differences. The growth model eliminates country‑specific fixed effects and, by transforming the data into year‑on‑year changes, removes the unit‑root components that are primarily responsible for serial correlation. The diagnostic results for this specification are presented in Table 3 (Panel B). The Breusch‑Godfrey test now fails to reject the null of no serial correlation for internet usage (p = 0.736) and patent applications (p = 0.424); only mobile subscriptions retain a significant lag‑dependence (p < 0.001), likely due to the exceptionally rapid saturation dynamics of mobile markets in BRICS countries. The RESET test no longer signals misspecification in the mobile and internet equations (p = 0.459 and p = 0.402, respectively), though the patent equation yields a borderline p‑value of 0.042, which does not survive a conservative Bonferroni correction. Heteroskedasticity also becomes less pervasive: the Breusch‑Pagan test fails to reject for patent applications (p = 0.087) but still identifies heteroskedastic disturbances in the mobile and internet equations (p < 0.001). Normality of the residuals remains strongly rejected in all cases, a finding that is asymptotically inconsequential given the moderately large number of cross‑sectional units (N = 41) and time periods (T = 29) [88].

Collectively, these diagnostic tests demonstrate that the growth specification alleviates the most critical assumption violations, particularly those related to serial correlation and functional form. However, because some heteroskedasticity and non‑normality persist, conventional standard errors are still unreliable. We therefore employ Driscoll‑Kraay standard errors (DK‑SE), which are robust to general forms of heteroskedasticity, cross‑sectional dependence, and autocorrelation [116]. Table 4 reports the DID coefficient estimates from both the level (Panel A) and the growth specifications (Panel B), alongside their conventional and Driscoll‑Kraay standard errors.

**Table 4:** DID coefficients with conventional and Driscoll‑Kraay standard errors from both the level and growth models.

Panel A: DID coefficients with conventional (Level) standard errors					
	**Coef**	**Conv SE**	**Conv p**	**DK SE**	**DK p**
LnMobCell	–1.0486	0.0903	0.0000	0.0116	0.0000
LnInt	–0.6137	0.0554	0.0000	0.0033	0.0000
LnPat	–0.1732	0.0798	0.0302	0.0038	0.0000
**Panel B: DID coefficients with Driscoll‑Kraay standard errors**					
	**Coef**	**Conv SE**	**Conv p**	**DK SE**	**DK p**
LnMobCell	−0.0546	0.0689	0.4277	0.0150	0.0003
LnInt	−0.2139	0.0605	0.0004	0.0208	0.0000
LnPat	−0.0669	0.0895	0.4548	0.0155	0.0000

The results are remarkably consistent across specifications. In the level model, the DID coefficient for mobile cellular subscriptions is –1.0486 (DK‑SE = 0.012, p < 0.001), for internet usage it is –0.6137 (DK‑SE = 0.003, p < 0.001), and for patent applications it is –0.1732 (DK‑SE = 0.004, p < 0.001). The growth model, which examines annual changes rather than levels, confirms the same directional pattern: the DID estimate for mobile subscriptions is –0.055 (DK‑SE = 0.015, p = 0.0003), for internet usage –0.214 (DK‑SE = 0.021, p < 0.001), and for patents –0.067 (DK‑SE = 0.016, p < 0.001). While the magnitudes differ—as expected when comparing a level with a growth rate—the negative sign and strong statistical significance are preserved throughout. This robustness triangulation strongly supports the conclusion that OECD countries experienced a post‑2010 relative slowdown in technology adoption and innovation activity compared with BRICS economies, and that this finding is not an artifact of the chosen functional form or of neglected dynamics.

To quantify the marginal contribution of the treatment indicator, we computed partial R² values for the DID interaction term in each of the three baseline TWFE models (see Table 5). The partial R² (DID estimator) is the unique proportion of variance in the dependent variable explained by the interaction term, after accounting for all other variables and fixed effects in the model [117]. The partial R² estimates paint a nuanced picture of how much the post‑2010 divergence between OECD and BRICS countries uniquely contributes to the variation in each outcome, over and above what is already explained by income levels, urbanization, foreign investment, and unemployment. For mobile cellular subscriptions and internet usage, the interaction term accounts for roughly 7.5% of the within‑country changes across time, a modest but clearly discernible share. This tells us that the differential growth trajectories of the two blocs—faster expansion in BRICS and a relative slowdown in OECD—genuinely matter for understanding the digital catch‑up, even after we strip out the influence of the usual economic drivers. In contrast, the interaction term for patent applications adds almost nothing (less than 0.3%), implying that the post‑2010 innovation gap is already well‑captured by the control variables and that the timing of the treatment itself has little independent explanatory power. This stark difference reinforces the study’s central message: convergence is most visible in basic technology adoption, while innovation output remains far more entangled with deep‑seated structural conditions that do not shift quickly in response to a particular policy era.

**Table 5:** Partial R² for the DID interaction term.

Model components	R²_full	R²_reduced	Partial R²
LnMobCell	0.392	0.3185	0.0735
LnInt	0.3051	0.2285	0.0766
LnPat	0.3436	0.3408	0.0029

#### 4.2.3. Placebo test.

To assess the credibility and robustness of the DID identification strategy and further mitigate concerns about unobserved confounding factors, a series of placebo tests are conducted for all three key outcome variables, mobile cellular subscriptions, internet usage, and patent applications. Specifically, we randomly assign the policy adoption status (treatment) and its implementation timing to countries, thereby constructing a pseudo-treatment variable. This variable is then substituted for the actual policy variable in the baseline regression for re-estimation. We repeat this randomization procedure 500 times. Figs 4-6 presents the kernel density distribution of the estimated coefficients from these placebo regressions. The distribution of the placebo coefficients is centered around zero and approximately follows a normal distribution, with the vast majority being statistically insignificant.

1. **Placebo test for mobile cellular subscription**

Based on the placebo test results for mobile cellular subscription shown in Fig 3, the distribution of estimated coefficients across placebo iterations centers around zero, with the majority of observations falling within a tight range close to the null effect. The kernel density plot indicates that the placebo coefficients are concentrated near zero, with a smooth, approximately symmetric density curve, suggesting that the placebo estimates do not systematically deviate from zero. The observed treatment effect from the actual DID model lies outside the high-density region of the placebo distribution, as indicated by its position relative to the plotted density mass.

**Fig 3:**
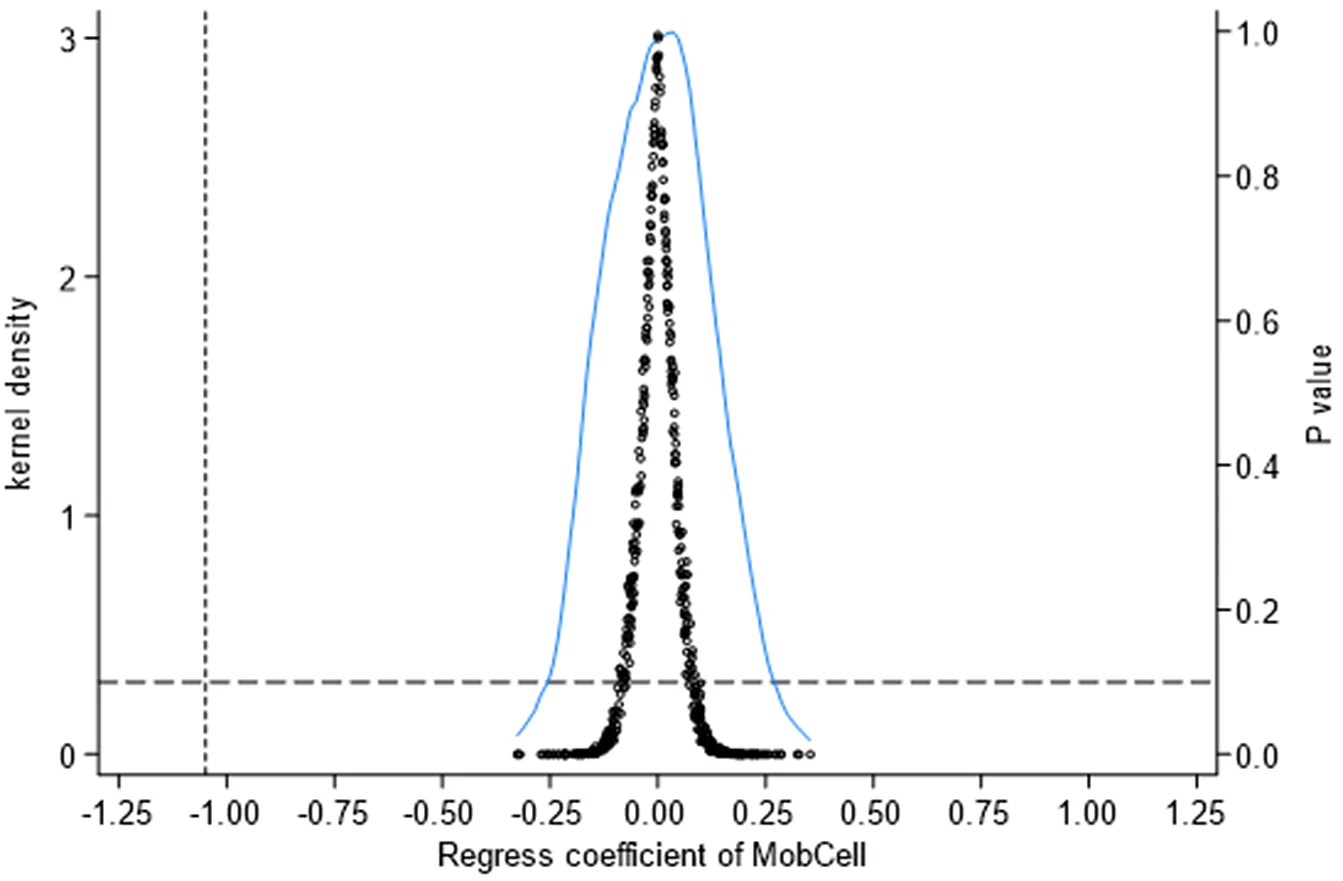
Placebo test result for mobile cellular subscription.

The p-value associated with the actual estimate is low, implying that the probability of observing an effect of such magnitude under the null hypothesis of no treatment effect is minimal when compared to the placebo distribution. This pattern supports the robustness of the main DID findings, as it suggests that the estimated impact of mobile cellular subscription on technological adoption is unlikely to be the result of random chance or unobserved temporal trends. The absence of significant placebo effects across the simulated iterations reinforces the credibility of the research design and bolsters confidence in the conclusion that Mobile Cellular Subscription significantly differs between treated and control groups in the post-treatment period.

The placebo test thus validates the parallel trends assumption underlying the DID approach and indicates that the identified relationship is not an artifact of spurious correlation or unmodeled confounding. These results strengthen the empirical basis for deriving policy implications regarding the role of mobile cellular infrastructure in shaping technological progress and stagnation across OECD and BRICS economies.

2. **Placebo test for internet usage**

Fig 4 presents the results of the placebo test for the key independent variable, internet usage. The kernel density plot illustrates the distribution of regression coefficients generated from a series of falsification tests, where the treatment timing is artificially randomized. The density curve is sharply peaked and centered very close to zero, with the vast majority of simulated coefficients concentrated in a narrow band between approximately −0.2 and +0.2. This creates a pronounced, needle-like distribution that falls away rapidly towards the tails.

**Fig 4:**
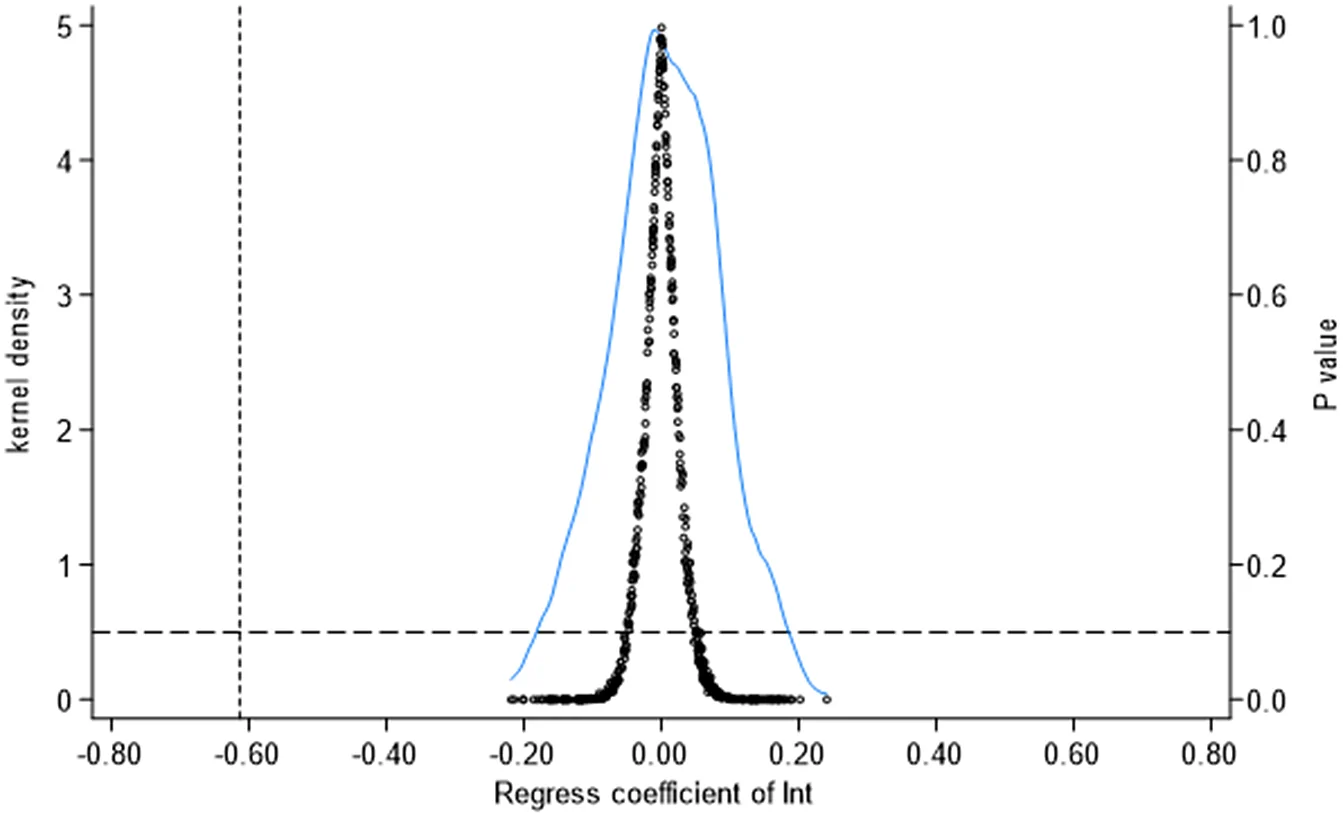
Placebo test result for internet usage.

Critically, the actual estimated coefficient from our difference-in-differences model, which captures the differential effect of internet usage growth in OECD versus BRICS nations post-2010, falls far outside this dense central cluster. Its location in the broader distribution is indicative of a statistically distinct effect that is not replicable under placebo conditions. The visual distance between the observed estimate and the placebo distribution’s core underscores that the identified relationship is unlikely to be a product of chance or latent temporal trends common to all countries.

This pattern provides strong empirical support for the robustness of our primary finding. The tight clustering of placebo coefficients around zero affirms that, in the absence of the true treatment, no systematic spurious effect is detected. Consequently, the test bolsters confidence in the validity of the parallel trends assumption for this variable and reinforces the conclusion that divergences in internet adoption have played a measurable and significant role in shaping the technological trajectory gap between the two groups of economies in the analysis period. This finding adds crucial credibility to subsequent discussions on policy interventions aimed at bridging digital divides.

3. **Placebo test for patent applications (Innovation)**

The robustness of the estimated treatment effect for patent applications is scrutinized through a falsification exercise, the results of which are visualized in the accompanying figure (Fig 5). The kernel density plot depicts the distribution of coefficients derived from placebo iterations, where the hypothetical treatment assignment is randomly shuffled across country groups and time. This distribution serves as an empirical benchmark for what the estimated effect would look like under the null hypothesis of no true causal relationship.

**Fig 5:**
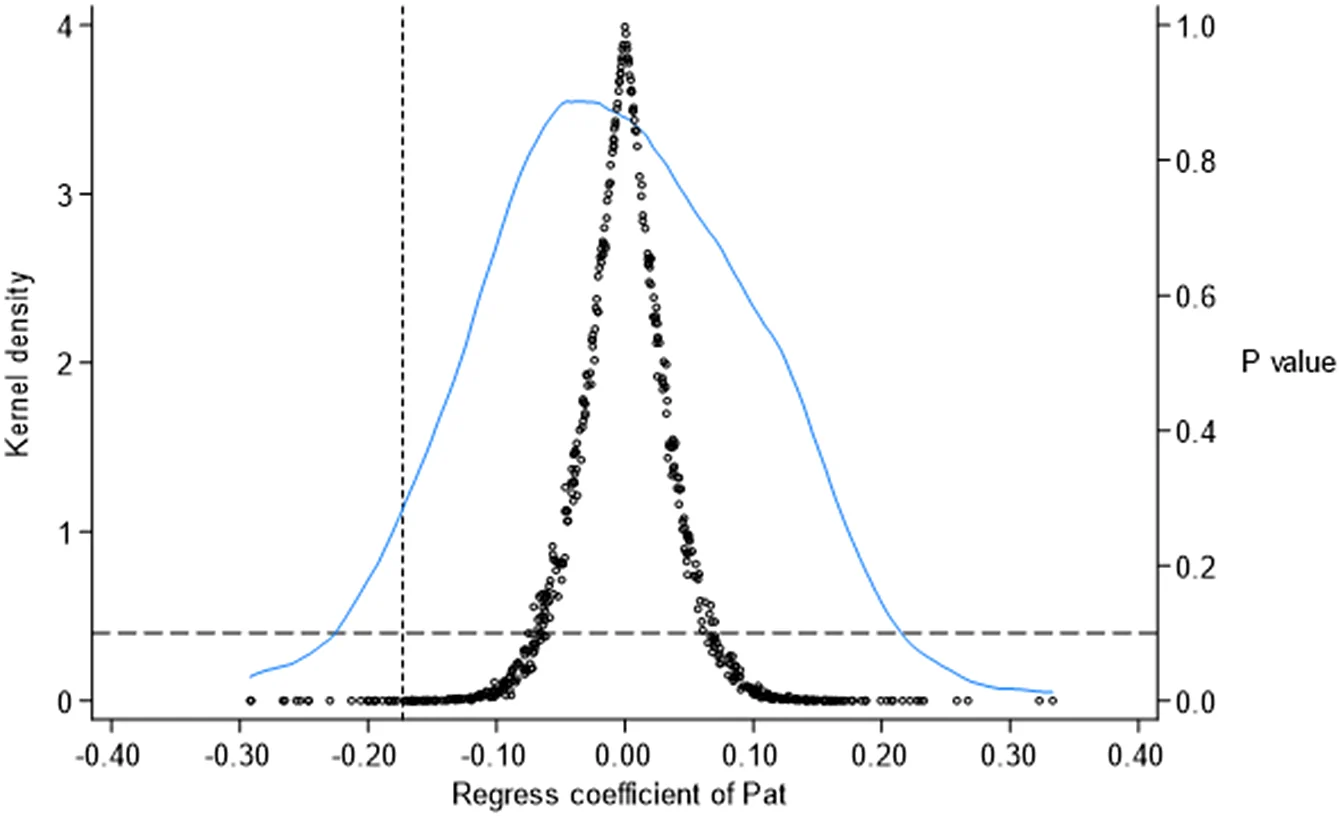
Placebo test result for patent applications.

A striking feature of the plot is the pronounced, unimodal peak of the density curve centered precisely at zero. This indicates that the vast majority of placebo estimates cluster tightly around a null effect, forming a steep, narrow distribution. The rapid decay of the density towards the tails suggests that large-magnitude coefficients are exceptionally rare in these randomized simulations. Visually, this creates a stark contrast between this concentrated “cloud of chance” and the actual estimated coefficient from our primary model, which is positioned distinctly to the right of this central mass.

The associated p-value for our observed coefficient is notably low, confirming that its magnitude falls within a percentile of the placebo distribution that would occur with very low probability if the true effect were zero. In substantive terms, this means the differential trajectory in patenting activity between OECD and BRICS economies following the treatment period is highly unlikely to be an artifact of spurious trends or unobserved confounders that affect all units similarly. The placebo test thus lends considerable credence to the parallel trends assumption for this specific metric of innovation output.

Consequently, this diagnostic fortifies our central inference regarding innovation divergence. It allows us to state with greater confidence that the identified gap in patent applications is not a statistical phantom but a robust signal of differing innovative capacities, potentially shaped by the distinct policy and institutional environments characterizing the two economic blocs post-2010. This finding sharpens the policy implications, directing attention toward the ecosystems that translate technological adoption into measurable innovative output.

#### 4.2.4. Parallel trend test.

1. **Panel trend test for mobile cellular subscriptions**

Fig 6 presents the dynamic DID estimates for mobile cellular subscriptions, comparing OECD and BRICS countries over the period 1995–2023, with the treatment year normalized to 2010. The coefficients capture the relative differences in mobile cellular adoption between the two groups at each point in time, conditional on controls for GDP per capita, urban population, unemployment rates, and FDI inflows.

**Fig 6:**
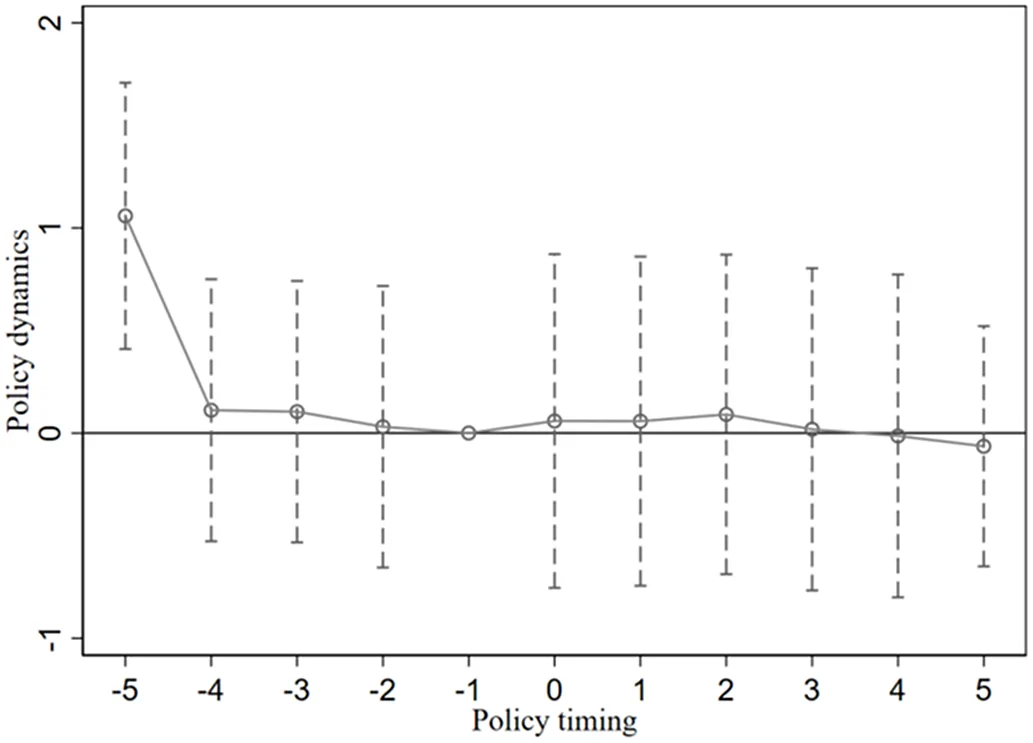
Parallel trend test for mobile cellular subscriptions.

During the pre-treatment period, the estimated coefficients are largely close to zero and statistically insignificant in the years immediately preceding the treatment (−4 to −1), providing support for the parallel trends assumption underlying the DID framework. Although a positive and statistically significant coefficient is observed at −5, this result likely reflects structural differences in initial adoption levels, with OECD countries exhibiting earlier leadership in mobile telephony. Importantly, the absence of statistically significant differences in the years just prior to 2010 suggests that the growth trajectories of mobile cellular subscriptions in OECD and BRICS countries were broadly comparable before the onset of the treatment period.

In the post-treatment period, the estimated coefficients remain small and statistically insignificant across all leads, fluctuating around zero. This pattern indicates that, following 2010, there is no evidence of a sustained or statistically meaningful divergence in mobile cellular subscription rates between OECD and BRICS countries. If anything, the slight decline in coefficients in later post-treatment years suggests a narrowing of the initial gap rather than an expansion.

Taken together, these findings point to a process of technological convergence in mobile cellular adoption between advanced and emerging economies. While OECD countries entered the period with a clear early-mover advantage, this advantage did not translate into a persistent post-2010 differential once mobile technology reached maturity and diffusion costs declined. The results are consistent with the DID empirical findings and the notion that mobile telephony represents a “catch-up” technology, where BRICS economies can rapidly expand adoption despite differences in income levels or labor market conditions.

2. **Panel trend test for internet usage**

Fig 7 illustrates the dynamic DID estimates for internet usage, comparing OECD and BRICS countries over the period 1995–2023, with 2010 designated as the treatment year. In the pre-treatment period (1995–2009), the estimated coefficients are modest in magnitude and statistically insignificant across most leads, fluctuating around zero. This pattern suggests that, despite differences in overall development levels, OECD and BRICS countries exhibited broadly similar trends in internet usage growth prior to 2010. The absence of systematic pre-treatment divergence provides empirical support for the parallel trends assumption and strengthens the credibility of the DID design.

**Fig 7:**
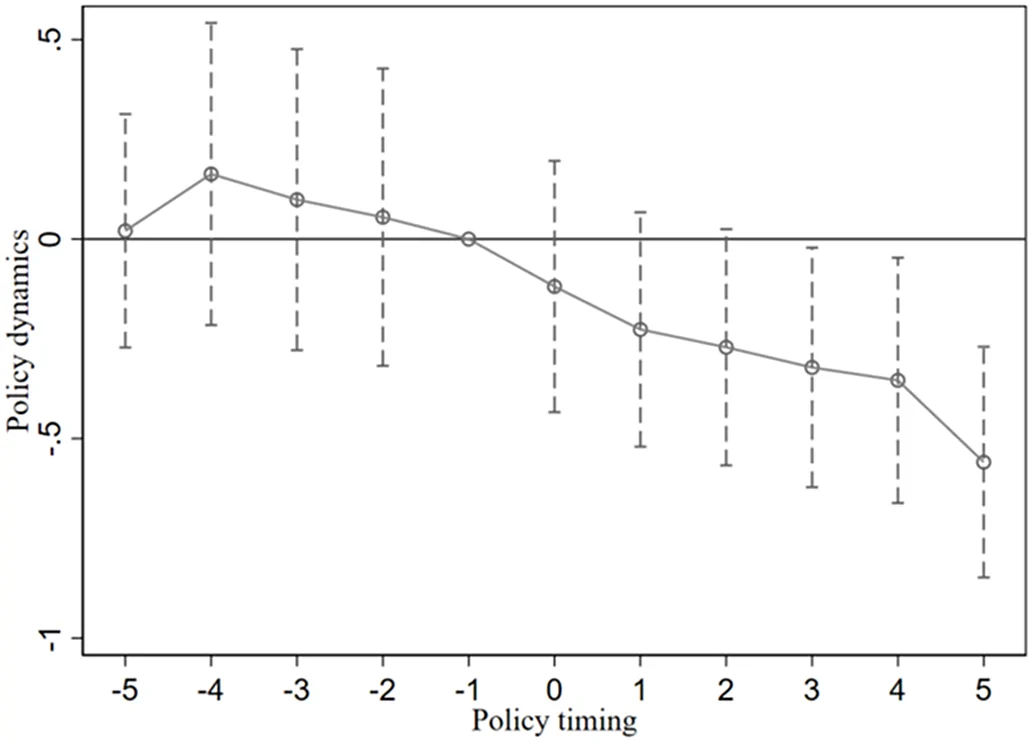
Parallel trend test for internet usage.

A marked shift emerges at the onset of the post-treatment period. From the treatment year onward, the estimated coefficients turn negative and display a clear downward trajectory. The magnitude of the negative coefficients increases over time, particularly from one to five years after treatment, indicating a growing divergence between OECD and BRICS countries in internet usage. Although some confidence intervals overlap zero in the immediate post-treatment years, the persistence and direction of the estimates point to a sustained relative slowdown in internet adoption in OECD countries compared to their BRICS counterparts.

This pattern contrasts sharply with the results observed for mobile cellular subscriptions and highlights an important distinction between basic technological adoption and more complex digital integration. While mobile telephony diffused rapidly across both groups, internet usage appears to be more sensitive to structural factors such as income levels, digital infrastructure, institutional quality, and complementary human capital. Even after controlling for urbanization, FDI inflows, GDP per capita, and unemployment, BRICS countries continue to experience faster growth in internet usage relative to their OECD counterparts, suggesting that deeper digital technologies are less amenable to leapfrogging.

From a policy perspective, these findings imply that the post-2010 period is characterized not by convergence but by digital divergence in internet usage. The results point to a form of technological stagnation in OECD countries with respect to advanced digital connectivity, despite progress in more basic technologies. This divergence may have long-term implications for innovation capacity, productivity growth, and participation in the digital economy, reinforcing the importance of targeted investments in broadband infrastructure, digital skills, and regulatory frameworks that support internet diffusion.

3. **Panel trend test for patent applications**

Fig 8 presents the dynamic DID estimates for patent applications, used here as a proxy for innovation activity, comparing OECD and BRICS countries over the period 1995–2023, with 2010 defined as the treatment year. In the pre-treatment period (1995–2009), the estimated coefficients fluctuate modestly around zero and are statistically insignificant across most leads. This pattern indicates that, despite clear differences in absolute innovation capacity, OECD and BRICS countries followed broadly similar trends in patent growth prior to 2010. The absence of systematic pre-treatment divergence provides support for the parallel trends assumption and suggests that subsequent differences can reasonably be attributed to post-treatment dynamics rather than pre-existing trend differences.

**Fig 8:**
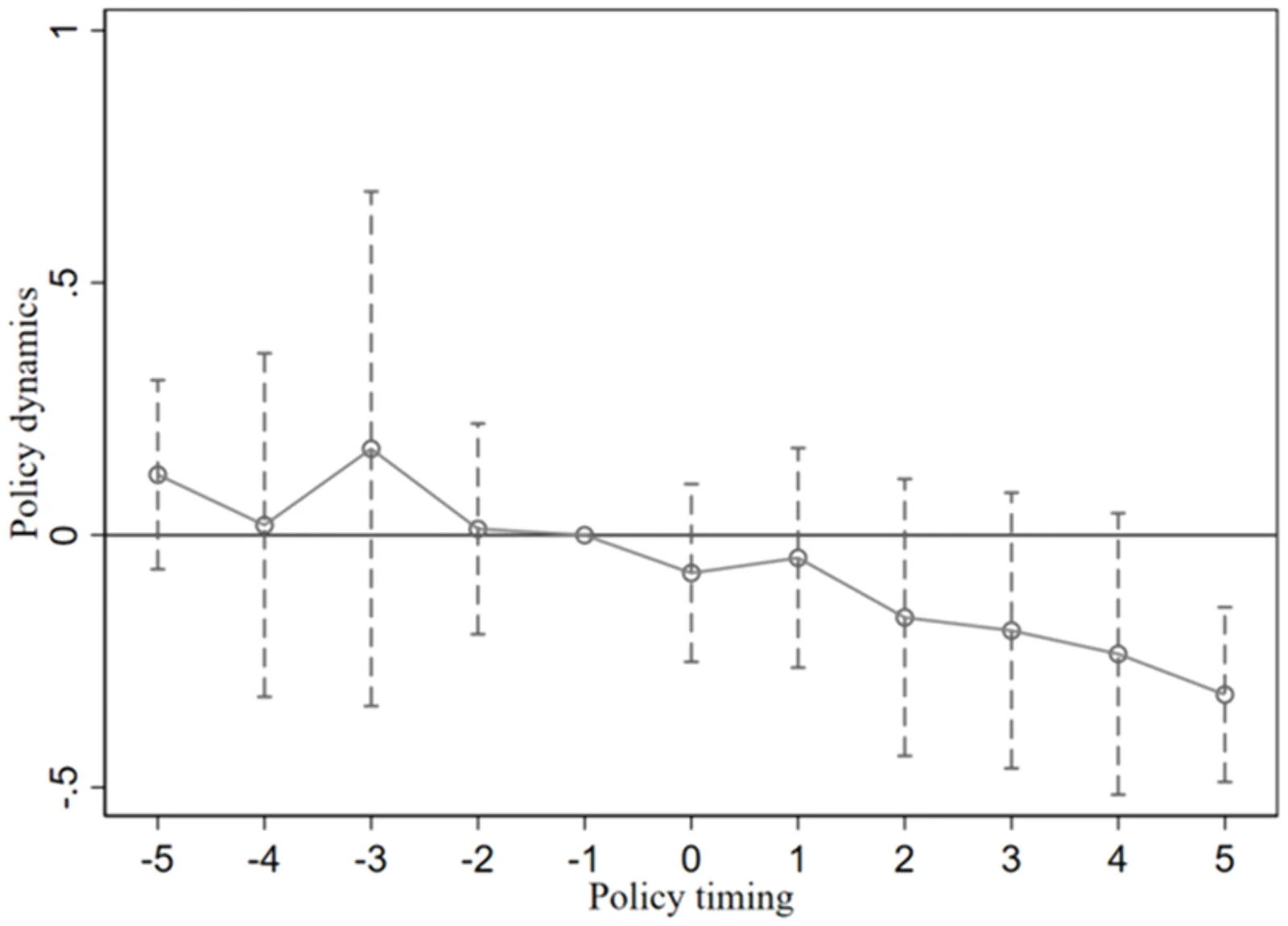
Parallel trend test for patent applications.

Following the treatment year, a gradual but persistent divergence emerges. Beginning in the early post-treatment period, the coefficients turn negative and continue to decline over time, with the magnitude of the negative estimates increasing in later years. Although the confidence intervals overlap zero in some post-treatment periods, the consistent downward trajectory points to a sustained relative slowdown in patenting activity in OECD countries compared to BRICS economies.

Unlike mobile cellular subscriptions, which exhibit convergence, and internet usage, which shows a more abrupt post-treatment divergence, the innovation gap measured through patent applications appears to widen slowly but steadily. This pattern suggests that innovation outcomes are shaped by deeper structural factors that evolve over longer horizons. Even after controlling for income levels, urbanization, labor market conditions, and foreign investment, BRICS countries maintain a growing advantage in knowledge production and formalized innovation.

From a policy perspective, these findings highlight the difficulty of closing innovation gaps through market forces alone. While emerging economies may rapidly adopt existing technologies, sustaining comparable levels of innovation requires complementary investments in research infrastructure, human capital, intellectual property institutions, and innovation ecosystems. The post-2010 divergence in patenting activity therefore reflects not short-term shocks, but longer-term institutional and structural constraints.

## 5. Conclusion

This paper examined differences in technological adoption and innovation between OECD and BRICS countries using the two-way fixed effects (TWFE) DID framework over the period 1995–2023, with particular attention to mobile cellular subscriptions, internet usage, and patent applications. By combining event-study analyses with placebo tests and alternative model specifications, the study provides a nuanced picture of how technological progress and innovation evolve across advanced and emerging economies.

The results reveal a clear distinction between patterns of technology adoption and innovation outcomes. For instance, the estimates for mobile cellular subscriptions reveal a negative and statistically significant treatment effect. Specifically, the coefficients of −1.0486 and −1.6301, obtained with and without the inclusion of control variables, indicate that OECD countries exhibit lower levels of mobile cellular subscriptions relative to BRICS countries. The results are robust at the 1% significance level, suggesting that this pattern is unlikely to be driven by random variation. Taken together, these findings point to a faster expansion of mobile telephony in BRICS economies over the study period, consistent with the rapid diffusion of relatively low-cost communication technologies in emerging markets.

A similar pattern emerges for internet usage. The estimated coefficients remain negative and statistically significant irrespective of model specification, with values of −0.6137 when controls are included and −0.8758 when they are excluded. These results imply that OECD countries, on average, display lower internet usage rates than BRICS countries during the post-treatment period. The consistency of the estimates across specifications strengthens the conclusion that BRICS economies have experienced more rapid growth in internet adoption. This trend likely reflects a combination of expanding urban populations, improvements in digital infrastructure, and growing demand for online services as these economies continue to develop.

The analysis of patent applications further reinforces the distinction. The estimated coefficients of −0.1732 and −0.9010, obtained with and without controls respectively, indicate that OECD countries record lower patenting activity relative to BRICS countries, although the magnitude of the difference is smaller than that observed for mobile and internet adoption. While the estimates remain statistically significant, the weaker effect suggests that convergence in innovation outcomes is more limited than in basic technology usage. This finding is consistent with the view that, although BRICS countries are increasingly engaging in innovative activities, innovation capacity remains more uneven and slower to adjust than adoption of widely diffused technologies.

The control variables provide additional context for understanding the dynamics of innovation and technology adoption. For instance, the coefficients for GDP per capita are all positive and statistically significant, indicating that economic prosperity is a strong predictor of technological adoption and innovation across all three measures. Higher GDP per capita correlates with increased mobile subscriptions (1.0710), internet usage (0.4827), and patent applications (0.5324), affirming the role of economic development in fostering technology and innovation.

The coefficients show mixed significance. A significant positive effect on patent applications (0.2091) indicates that higher unemployment may correlate with increased innovation, possibly due to a larger pool of available talent or shifts in labor towards more innovative sectors. However, the coefficients for mobile subscription (0.0952) and internet usage (0.0153) are less impactful, suggesting that unemployment does not have a strong direct relationship with mobile or internet adoption.

The coefficients for urban population show a positive but not statistically significant relationship for mobile cellular subscription (0.6055) and internet usage (0.2715), while it has a significant positive effect on patent applications (4.3330). This indicates that urbanization is a critical driver of innovation, particularly in terms of patenting activities, suggesting that more urbanized areas foster environments conducive to innovation.

The positive and statistically significant coefficients (0.0467 for mobile subscription and 0.0382 for internet usage indicate that increased FDI inflows are associated with higher mobile cellular subscriptions and internet usage. However, the negative coefficient for patent applications (−0.1079) suggests that while FDI may boost technology adoption, it might not directly translate into increased patenting, possibly indicating that foreign investments do not always lead to local innovation.

From a policy perspective, the findings suggest that while BRICS countries have made substantial progress in adopting widely diffused technologies and are increasingly active in innovation, sustaining long-term convergence with advanced economies requires more than rapid uptake alone. Policy efforts should therefore move beyond facilitating access to existing technologies toward strengthening the foundations of domestic innovation. This includes targeted investments in digital infrastructure, human capital development, and research institutions, alongside regulatory frameworks that encourage knowledge creation and diffusion. In addition, policies that leverage foreign direct investment as a complement to local innovation, rather than a substitute, can help embed technological capabilities within domestic firms. Given the role of urbanization in shaping technology adoption, ensuring that urban centers are equipped with adequate digital and innovation-supporting infrastructure is also critical. Overall, distinguishing between basic technology diffusion and deeper innovation capacity is essential for designing policies that translate short-term adoption gains into sustained productivity growth and technological upgrading.

Several boundaries of this study deserve mention. First, the dependent variables are macro‑level indicators that capture quantity rather than quality. Mobile subscriptions count SIM cards, not unique users, and internet usage does not reflect connection speed or the sophistication of online activities. Second, we purposely opted not to include fixed broadband subscriptions (ICT infrastructure) as a separate outcome because the technology was negligible in many BRICS economies during the early years of the panel [118,119] and is highly collinear with internet usage. However, we fully recognize that this choice narrows our digital infrastructure lens. Future research should adopt a more comprehensive ICT framework, potentially using composite indices such as the ITU ICT Development Index, or leveraging data on mobile and fixed broadband speeds, to test whether convergence patterns hold when one looks beyond simple access. Furthermore, our study does not test for treatment effect heterogeneity across countries or time. Thus, we suggest that future research could apply newer estimators (e.g.[120,121],) that are robust to heterogeneous treatment timing.

## Supporting information

S1 Data Excel file. This Excel file contains the values for all key variables examined in the study, including Urban population (UrbPop), Foreign direct investment inflow (FDI), GDP per capita (GDPpc), Unemployment rate (UnEmp), Mobile cellular subscriptions (MobCell), Internet usage (Int), Patent applications (Pat), Pre-treatment (1995–2009 = 0); Post-treatment (2010–2023 = 1), Treatment group (OECD = 1); Control group (BRICS 0).
(XLSX)

S1 Table Descriptive statistics – OECD and BRICS countries. (DOCX)

S1 Fig Trends in the control variables for OECD and BRICS countries, 1995–2023. (PNG)
