# Supplementary material for: Technological adoption and innovation in OECD and BRICS countries: A two‑way fixed effects difference‑in‑differences analysis
Source: PLoS One. 2026 Nov 4;21(11):e0347936. doi: 10.1371/journal.pone.0347936 (PMC13734478; doi:10.1371/journal.pone.0347936)
Supplement: Supplementary file 1 [file pone.0347936.s002.docx]

**S1 Table**. Descriptive statistics – OECD and BRICS countries.

| **Descriptive Statistics – OECD** | | | | | | | |
| --- | --- | --- | --- | --- | --- | --- | --- |
|  | **LnMobCell** | **LnInt** | **LnPat** | **LnGDPpc** | **LnUnEmp** | **LnUrbPop** | **LnFDI** |
| Mean | 4.2118 | 3.8162 | 7.1256 | 10.1101 | 1.8957 | 4.3223 | 6.0988 |
| Median | 4.6596 | 4.2767 | 7.2154 | 10.2651 | 1.8966 | 4.3562 | 6.0996 |
| Maximum | 5.1763 | 4.6230 | 12.8671 | 11.8034 | 3.2617 | 4.5869 | 6.5180 |
| Minimum | -1.6332 | 0.7332 | 2.0794 | 7.6883 | 0.5906 | 3.9244 | 1.0540 |
| Std. Dev. | 1.0758 | 0.9783 | 2.3874 | 0.8566 | 0.4819 | 0.1565 | 0.1588 |
| Skewness | -2.3516 | -1.5528 | 0.4027 | -0.7470 | 0.0609 | -0.6018 | -30.761 |
| Kurtosis | -2.3516 | 1.3262 | -0.0852 | 0.0669 | -0.2570 | -0.4093 | 975.613 |
| Jarque-Bera | 2412.32 | 496.07 | 28.5291 | 97.2906 | 3.5185 | 70.3134 | 41568 |
| Probability | 0.0000 | 0.0000 | 0.0000 | 0.0000 | 0.0172 | 0.0000 | 0.0000 |
| Sum | 4397.09 | 3984.13 | 7439.13 | 10554.91 | 1979.13 | 4512.46 | 63672 |
| Sum Sq. Dev. | 1207.02 | 998.31 | 5944.58 | 765.34 | 242.26 | 25.5440 | 26.2879 |
| Observations | 1044 | 1044 | 1044 | 1044 | 1044 | 1044 | 1044 |
| **Descriptive Statistics – BRICS** | | | | | | | |
|  | **LnMobCell** | **LnInt** | **LnPat** | **LnGDPpc** | **LnUnEmp** | **LnUrbPop** | **LnFDI** |
| Mean | 3.3180 | 2.8616 | 9.1835 | 8.2327 | 2.1206 | 4.0225 | 6.0985 |
| Median | 4.3591 | 3.0493 | 8.8904 | 8.5382 | 1.8596 | 4.1391 | 6.0979 |
| Maximum | 5.1298 | 4.5454 | 14.1708 | 9.6767 | 3.5107 | 4.4749 | 6.1151 |
| Minimum | -4.8343 | 0.6956 | 4.9273 | 5.9274 | 1.0999 | 3.2812 | 6.0896 |
| Std. Dev. | 2.1442 | 1.2776 | 2.0745 | 1.0019 | 0.6831 | 0.3777 | 0.0034 |
| Skewness | -1.5628 | -0.3182 | 0.6938 | -0.6387 | 0.8039 | -0.6566 | 0.9615 |
| Kurtosis | 1.7774 | -1.3488 | 0.2157 | -0.6023 | -0.6499 | -1.0135 | 2.9171 |
| Jarque-Bera | 78.1093 | 13.4386 | 11.9130 | 12.0506 | 18.1695 | 16.6234 | 73.7511 |
| Probability | 0.0000 | 0.0012 | 0.0026 | 0.0024 | 0.0001 | 0.0002 | 0.0000 |
| Sum | 481.10 | 414.93 | 1331.60 | 1193.74 | 307.48 | 583.26 | 884.28 |
| Sum Sq. Dev. | 662.06 | 235.04 | 619.70 | 144.53 | 67.19 | 20.54 | 0.00 |
| Observations | 145 | 145 | 145 | 145 | 145 | 145 | 145 |
